# Nicotinamide mononucleotide and related metabolites induce disease resistance against fungal phytopathogens in Arabidopsis and barley

**DOI:** 10.1038/s41598-017-06048-8

**Published:** 2017-07-25

**Authors:** Akihiro Miwa, Yuji Sawada, Daisuke Tamaoki, Masami Yokota Hirai, Makoto Kimura, Kazuhiro Sato, Takumi Nishiuchi

**Affiliations:** 1 Division of Life Science, Graduate School of Natural Science and Technology, Kanazawa University, Kakuma-cho, Kanazawa, Ishikawa 920-1192 Japan; 2 RIKEN Center for Sustainable Resource Science, 1-7-22 Suehiro-cho, Tsurumi-ku, Yokohama, Kanagawa 230-0045 Japan; 3 Division of Functional Genomics, Advanced Science Research Centre, Kanazawa University, 13-1 Takara-machi, Kanazawa, Ishikawa 920-0934 Japan; 4 Division of Molecular and Cellular Biology, Graduate School of Bioagricultural Sciences, Nagoya University, Furo-cho, Chikusa, Nagoya, Aichi 464-8601 Japan; 5 Institute of Plant Science and Resources, Okayama University, Chuo 2-20-1, Kurashiki, Okayama 710-0046 Japan; 6 Present Address: Graduate School of Science and Engineering, University of Toyama, 3190 Gofuku, Toyama-shi, Toyama 930-8555 Japan

## Abstract

Nicotinamide mononucleotide (NMN), a precursor of nicotinamide adenine dinucleotide (NAD), is known to act as a functional molecule in animals, whereas its function in plants is largely unknown. In this study, we found that NMN accumulated in barley cultivars resistant to phytopathogenic fungal *Fusarium* species. Although NMN does not possess antifungal activity, pretreatment with NMN and related metabolites enhanced disease resistance to *Fusarium graminearum* in Arabidopsis leaves and flowers and in barley spikes. The NMN-induced *Fusarium* resistance was accompanied by activation of the salicylic acid-mediated signalling pathway and repression of the jasmonic acid/ethylene-dependent signalling pathways in Arabidopsis. Since NMN-induced disease resistance was also observed in the SA-deficient *sid2* mutant, an SA-independent signalling pathway also regulated the enhanced resistance induced by NMN. Compared with NMN, NAD and NADP, nicotinamide pretreatment had minor effects on resistance to *F. graminearum*. Constitutive expression of the *NMNAT* gene, which encodes a rate-limiting enzyme for NAD biosynthesis, resulted in enhanced disease resistance in Arabidopsis. Thus, modifying the content of NAD-related metabolites can be used to optimize the defence signalling pathways activated in response to *F. graminearum* and facilitates the control of disease injury and mycotoxin accumulation in plants.

## Introduction

Plants protect themselves from pathogen attack by activating a variety of defence responses, including the production of antimicrobial compounds and proteins^1^. Phytoalexins and phytoanticipins are antimicrobial compounds that accumulate in plant tissues with and without phytopathogen attack, respectively. Plants secrete antimicrobial proteins such as thionin to prevent the entry of pathogens into cells^2^. These antimicrobial substances directly inhibit the growth of pathogens on plant surfaces, whereas plant activators induce the innate immune response rather than possessing antimicrobial properties themselves. SA analogues such as 2,6-dichloroisonicotinic acid (INA) and benzo (1,2,3) thiadiazole-7-carbothionic acid S-methyl ester (BTH) hyperactivate the SA-dependent immune response. Vernooij *et al*.^3^ showed that INA acts via the systemic acquired resistance (SAR) signal transduction pathway. Similarly, BTH has been shown to enhance disease resistance against *Pseudomonas syringae* pv. tomato strain DC3000 (*Pst*DC3000) and induce the expression of SAR genes^4, 5^. These compounds do not possess antimicrobial activities and are referred to as plant defence activators. In addition, BTH and SA positively regulate disease resistance against fungal pathogens in Arabidopsis^6^ and wheat^7^.

Some plant-derived metabolites are also known to act as plant activators^8^. These natural compounds are therefore potential safe agents that could be used to control disease injury caused by a broad range of phytopathogens. It has been previously reported that the extracellular nicotinamide adenine dinucleotide (NAD) and nicotinamide adenine dinucleotide phosphate (NADP) caused an accumulation of SA and the induction of pathogenesis-related (*PR*) genes via the Ca^2+^-dependent signalling pathway, enhancing resistance to *Pseudomonas syringae* pv. *maculicola* ES 4326 (*Psm* ES4326)^9^. NAD and NADP are known to act as cofactors in various redox reactions as well as to be involved in biotic and abiotic stress responses^10, 11^. In addition, manipulation of certain NAD biosynthesis genes in plants has been shown to affect redox signalling through the production of reactive oxygen species (ROS) and pyridine alkaloids, resulting in activation of disease resistance to phytopathogens including *Pst*DC3000 via the SA-dependent signalling pathway^12, 13^. In animals, NMN, a precursor of NAD biosynthesis, is known to exert anti-ageing effects via sirtuin (SIRT) genes encoding NAD-dependent protein deacetylases^14^. However, the biological function of NMN in plants is largely unknown.

*Fusarium* species such as *F. graminearum*, *F. culmorum*, and *F. asiaticum* are hemibiotrophic or necrotrophic fungal phytopathogens and causal agents of head blight in wheat and barley^15^. These fungi often produce trichothecene mycotoxins, which are known to act as translation inhibitors in eukaryotic cells^16^. DON (deoxynivalenol)-producing *F. graminearum* exists worldwide, whereas NIV (nivalenol)-producing *F. asiaticum* is found only in Asia^16^. Under high humidity conditions, these *Fusarium* species can easily infect the flowers (spikes) of wheat and barley^15^. Contamination of grains with trichothecenes is often found worldwide, threatening human and animal health^17^. However, commercial cereal cultivars showing strong resistance to *Fusarium* diseases are not yet available^17^. Therefore, wheat and barley plants are treated at the flowering stage with fungicides to decrease yield and quality losses. However, fungicide residues on crops are not beneficial to human or animal health. In addition, fungicide-resistant *Fusarium* strains are frequently reported^18^. Safe and effective agents are therefore required for the control of *Fusarium* disease in cereal crops.

We previously identified many barley cultivars showing a Fusarium head blight (FHB) resistance phenotype using cut-spike methods^19, 20^. Among these cultivars, Maja and Sirius O.525 exhibited high FHB resistance. We therefore performed a comparative analysis of the metabolite profiles of these resistant cultivars vs. FHB-susceptible cultivars (Turkey 45 and H.E.S.4). Some metabolites, including NMN, accumulated in the FHB-resistant lines. NAD is known to play roles in plant defence responses against bacterial pathogens^13^. To estimate the effect of NMN on disease resistance to *F. graminearum*, we used Arabidopsis model plants, which are known to be susceptible to this pathogen. Exogenous application of NMN activated defence signalling and resulted in enhanced disease resistance against *F. graminearum* in Arabidopsis. Furthermore, exogenous application of NMN also significantly decreased disease development and DON production by *F*. *graminearum* in an FHB-susceptible barley cultivar. Thus, NMN could be useful in controlling disease injury and mycotoxin contamination by *Fusarium* species in various plant species.

## Results and Discussion

### NMN accumulation in FHB-resistant barley cultivars

We previously identified many barley cultivars that exhibited an FHB-resistant phenotype^19, 20^. Two 2-row cultivars (U389; Maja and U121; Sirius O-525) showed high FHB resistance compared with susceptible cultivars (Fig. S1a,b). We also performed a comparative analysis of the metabolite profiles of these FHB-resistant and FHB-susceptible (T615; Turkey 45) 2-row cultivars. NMN was found to be enriched in the uninoculated barley spikes of the two resistant cultivars compared with the susceptible cultivar (Fig. S1c). At 2 days post inoculation, the NMN contents had not significantly changed in any of the three cultivars (data not shown). NMN is a precursor of NAD, which functions as a cofactor for many enzymes involved in the catalysis of various metabolic reactions^21^. A similar accumulation pattern was not observed for other NAD-related metabolites (NaMN: nicotinic acid mononucleotide, NA: nicotinate, NIC: nicotinamide; data not shown). Unfortunately, NAD(H) and NADP(H) were not quantified in this system. Zhang & Mou^9^ reported that extracellular NAD induced *PR* gene expression and resistance against the bacterial pathogen *P. syringae* in *Arabidopsis thaliana*. These results suggest that NMN is also involved in plant disease resistance against *Fusarium* species. It has also been reported that the amount of NAD significantly increased in barley leaves inoculated with the biotrophic fungal pathogen *Erysiphe graminis*, the causal organism of powdery mildew^22^. These findings suggest that NAD biosynthesis is likely involved in disease resistance against a broad range of phytopathogens in barley. The NAD(H) and NADP(H) pools function as coenzymes for the various redox reactions. NMN is a precursor of not only NAD(H) and NADP(H) but also antimicrobial pyridine alkaloids^23^. These facts imply that NMN controls plant disease more effectively than NAD or NADP.

In addition, we examined the antifungal activity of NMN against *F. graminearum* (Fig. S1d). For this purpose, the inhibitory effect on mycelial growth was investigated. We used the 3-(4,5-di-methylthiazol-2-yl)-2,5-diphenyltetrazollium bromide (MTT) method to analyse the growth of *F. graminearum* ^2, 24^. We found that *F. graminearum* mycelium growth was not inhibited by NMN (Fig. S1d). These results indicate that NMN does not have antifungal activity against *F. graminearum*.

As stated above, a high concentration (>1 mM) of extracellular NAD activates defence signalling events, including the induction of *pathogenesis-related 1* (*PR1*) expression^9^. To examine whether NMN can activate defence signalling in Arabidopsis plants, we investigated the expression of the SA-responsive *PR1* gene, the SA biosynthesis gene *ICS1*, and the JA/ET-responsive *plant defensin 1.2a* (*PDF1.2a*) gene by RT-qPCR (Fig. S2a, S2b, S2c). Plants were treated with NMN at a concentration of 0.3 mM, which was sprayed onto the surface of rosette leaves. The leaves were then harvested at different time points (0, 6, 24, and 48 h) after spraying. The mRNA levels of the *PR1* gene increased at 6 h after NMN treatment and then decreased (Fig. S2a). Correspondingly, *ICS1* gene expression was also transiently increased by NMN treatment (Fig. S2b). On the other hand, NMN treatment did not induce expression of the *PDF1.2a* gene (Fig. S2c). These results suggest that NMN transiently activates the SA-dependent signalling pathway in Arabidopsis leaves. It has previously been reported that SA-dependent signalling positively regulates plant disease resistance against *F. graminearum* ^6, 7^.

### NMN activates disease resistance against *F. graminearum* in Arabidopsis leaves and flowers

**Figure 1:**
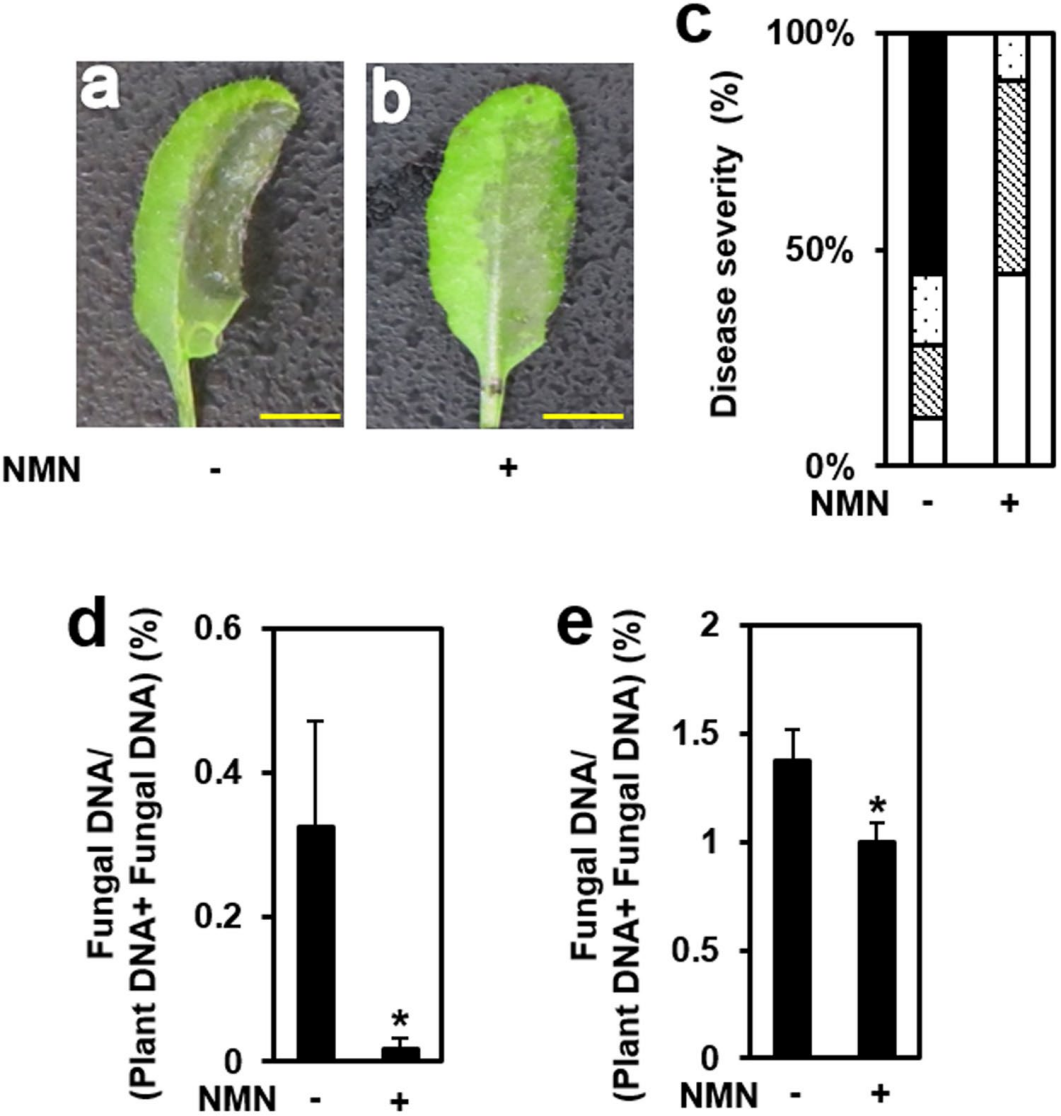
NMN induced disease resistance against *F. graminearum* in Arabidopsis. (**a,b**) Water or NMN (0.3 mM) was sprayed onto the surface of Arabidopsis rosette leaves and flower buds prior to incubation for 6 h. Conidia solutions (1 × 10^5^ conidia/ml) of *F. graminearum* were then injected into leaves and sprayed onto flowers. Plants inoculated with *F. graminearum* were kept under high humidity conditions. Representative photographs of *F. graminearum*-inoculated leaves with water (**a**) or NMN pretreatment (**b**). Scale bars (**a,b**): 1 cm. (**c**) Disease severity of *F. graminearum*-inoculated leaves with and without NMN pretreatment. Disease severity was evaluated by observations of symptoms on inoculated leaves 3 days after inoculation (n = 18). Open box: normal, cross-hatched box: colour change, dot box: partial aerial mycelium, closed box: expanded aerial mycelium. (**d**) *F. graminearum* gDNA was measured by qPCR in inoculated leaves. Error bars represent the standard deviation (n = 3) (Student’s t-test *P < 0.05). (**e**) *F. graminearum* gDNA was measured by qPCR in inoculated flower buds. Error bars represent the standard deviation (n = 3) (Student’s t-test *P < 0.05).

We examined the effect of NMN pretreatment on disease resistance against *F. graminearum* in Arabidopsis plants. Six hours after spraying NMN at various concentrations onto leaf surfaces, conidia solutions of *F. graminearum* were infiltrated into the leaves using a needleless syringe (Fig. S3). When water-pretreated Arabidopsis leaves were inoculated with *F. graminearum* conidia, severe disease symptoms and extended hyphae were observed (Fig. 1a,c). Pretreatment with 0.3 mM NMN alleviated the disease symptoms of leaves inoculated with *F. graminearum* (Fig. 1b,c, Fig. S3). However, similar effects were not observed in 3 mM NMN-pretreated Arabidopsis leaves (Fig. S3). We also quantified the *F. graminearum* genomic DNA in inoculated leaves by qPCR (Fig. 1d). The amount of fungal genomic DNA in NMN-pretreated leaves was significantly decreased compared with that in water-pretreated leaves (Fig. 1d). To examine the effects of NMN on *Fusarium* resistance in Arabidopsis flowers, an NMN solution was sprayed onto flowers, and 6 h after the NMN treatment, the flowers were inoculated with *F. graminearum* conidia via spraying. The fungal genomic DNA derived from *F. graminearum* was also decreased in NMN-treated flowers (Fig. 1e). The inoculated flower samples used for gDNA isolation contained uninfected tissues such as inflorescence stems. Therefore, the difference between water- and NMN-pretreated flowers was relatively small compared with that of leaves. Thus, pretreatment with NMN enhanced disease resistance against *F. graminearum* in Arabidopsis leaves and flowers.

Similarly, it has been reported that exogenous application of NAD and NADP enhanced disease resistance against a bacterial pathogen, *Psm* ES4326^9^. Pétriacq *et al*.^25^ reported that higher NAD contents in *nadC*-overexpressing plants with the addition of quinolinate activated the plant immune response and resulted in disease resistance against a virulent bacterial pathogen, *Pst-AvrRpm1*. The *nadC* gene derived from *E. coli* catalyses the conversion of quinolinate to NaMN^25^. Therefore, the accumulation of NAD and related metabolites induces disease resistance against bacterial phytopathogens. However, a higher concentration (3 mM) of NMN did not induce disease resistance to *F. graminearum* (Fig. S3). The optimal concentration of NAD-related metabolites may differ depending on the type of phytopathogen. In addition, different signalling events may occur upon treatment with a lower concentration (0.3 mM) of NMN.

**Figure 2:**
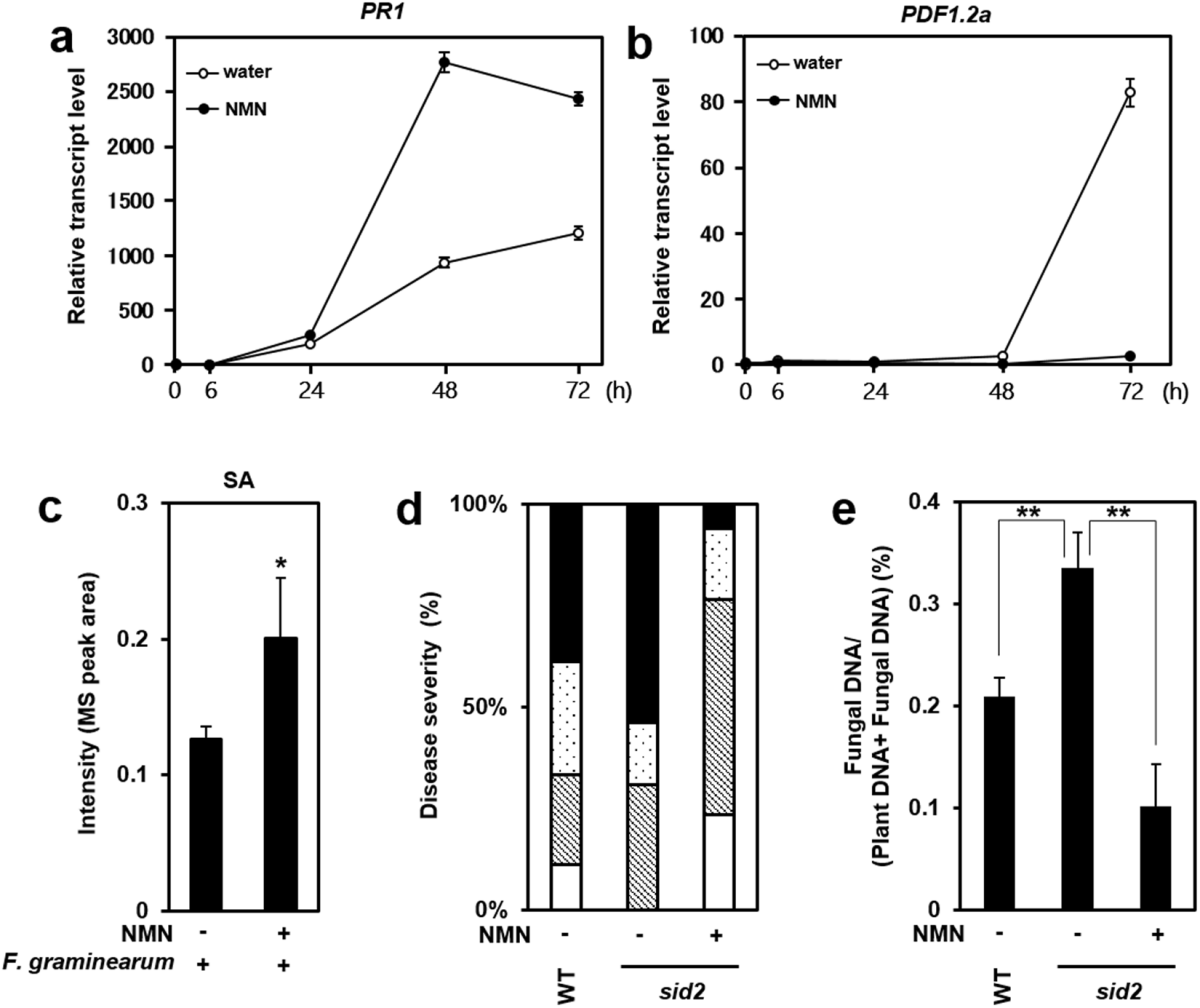
Involvement of the SA signalling pathway in *F. graminearum*-infiltrated Arabidopsis leaves with and without NMN pretreatment. (**a,b**) RT-qPCR analysis of *PR1* and *PDF1.2a* mRNA expression in *F. graminearum*-inoculated WT plants with and without NMN pretreatment. Six hours after NMN pretreatment (time point 0), leaves were injected with *F. graminearum* (n = 18). Plants were incubated under high humidity conditions and harvested at 0, 6, 24, 48 and 72 h. *ACTIN2/8* (*Act2/8*) was used as the reference gene. The data represent the average of all samples, and error bars represent the standard deviation (n = 3). *ACTIN2/8* (*Act2/8*) was used as the reference gene. Each value is shown as fold change (each sample vs 0 h of water treatment). Error bars represent the standard deviation (n = 3). (**c**) Accumulation of salicylic acid (SA). (Student’s t-test *P < 0.05) (**d,e**) NMN induces disease resistance against *F. graminearum* via the salicylic acid (SA)-independent signalling pathway. NMN (0.3 mM) was sprayed onto the surface of *sid2-2* rosette leaves prior to incubation for 6 h. Six hours after NMN treatment, conidia solutions (1 × 10^5^ conidia/ml) of *F. graminearum* were injected into leaves. (**d**) The disease severity was evaluated by the disease symptoms of inoculated leaves 3 days after inoculation (n = 18). Open box: normal, cross-hatched box: colour change, dot box: partial aerial mycelium, closed box: expanded aerial mycelium. (**e**) *F. graminearum* DNA was measured by qPCR. Error bars represent the standard deviation (n = 3) (Student’s t-test **P < 0.01).

**Figure 3:**
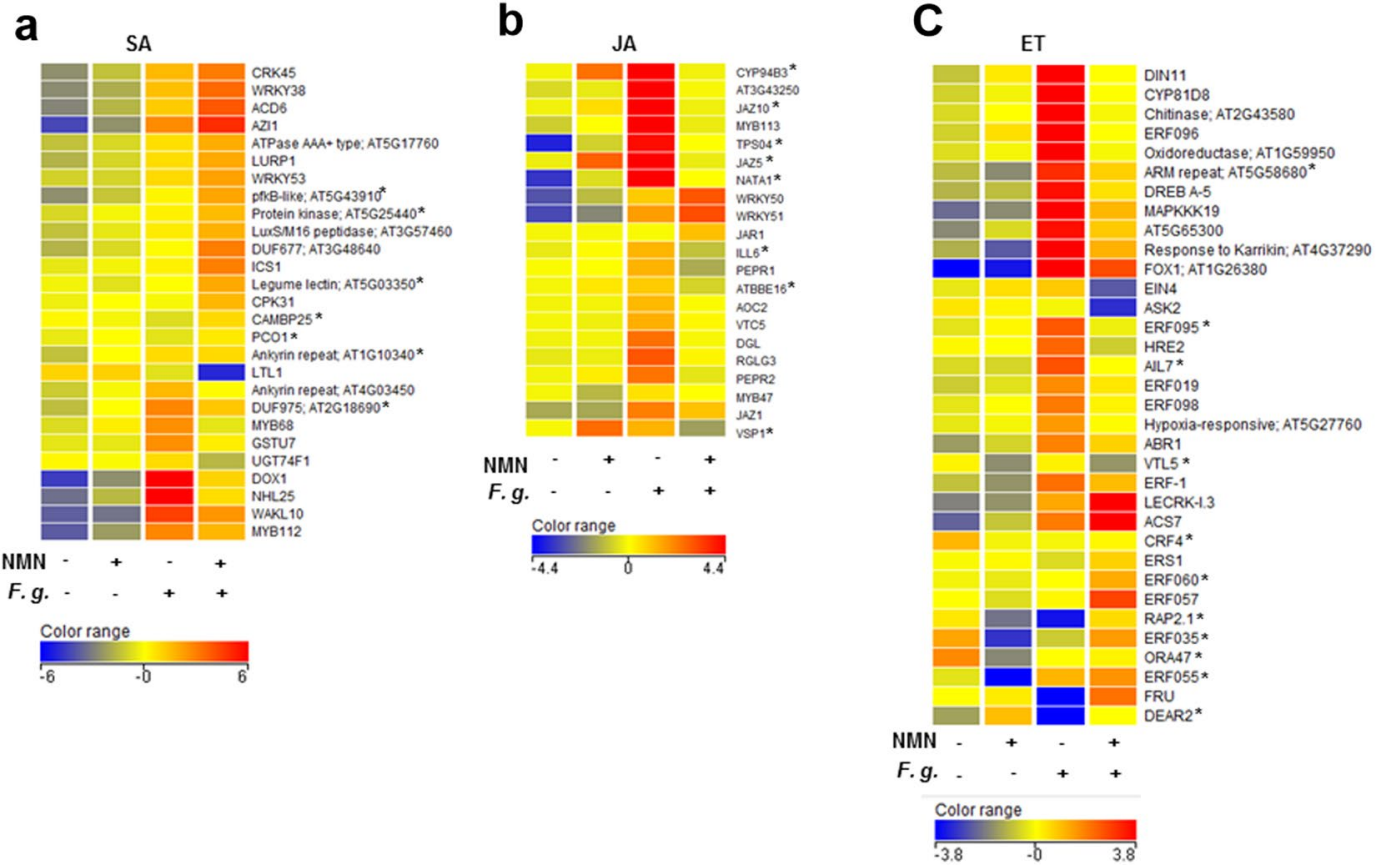
Hierarchical clustering of NMN-regulated genes with or without inoculation of *F. graminearum* in SA (**a**), JA (**b**), and ET (**c**) categories. SA-, JA- and ET-related genes were selected from NMN-induced or NMN-suppressed genes (FC > 2, P < 0.05) with or without inoculation, based on their GO term names. Then, hierarchical clustering analysis was performed using GeneSpring GX ver.12.5. The level of the bar colour indicates the magnitude of higher expression (red colour) or lower expression (blue colour) of each gene after normalization. *F.g*.; inoculated with *Fusarium graminearum*. Asterisk: Benjamini and Hochberg FDR was greater than 0.05.

As stated above, NMN application transiently induced expression of the *PR1* gene in Arabidopsis leaves. Figure 2a shows that expression of the *PR1* gene was up-regulated 48 h after inoculation in NMN-pretreated leaves compared with water-pretreated leaves. By contrast, induction of the *PDF1.2a* gene by *F. graminearum* inoculation was significantly suppressed in NMN-pretreated leaves (Fig. 2b). In addition, the SA biosynthesis gene *ICS1* was also up-regulated by NMN pretreatment in *F. graminearum*-inoculated leaves (Fig. S4). These results suggest that pretreatment with NMN enhanced the SA-dependent signalling pathway but completely suppressed the JA/ET signalling pathway in leaves inoculated with *F. graminearum*. Correspondingly, it has previously been reported that resistance to *F. graminearum* was positively and negatively regulated by SA and JA/ET signalling, respectively^6, 26^. The accumulation of SA in *F. graminearum*-inoculated leaves increased with NMN pretreatment (Fig. 2c). It is likely that this enhanced accumulation of SA mediated by NMN pretreatment led to enhanced resistance to *F. graminearum*. We next analysed NMN-induced resistance against *F. graminearum* in the SA-deficient *salicylic acid induction-deficient 2* (*sid2*) mutant. Although the *sid2* mutant exhibited a more susceptible phenotype than the wild type (WT), NMN-induced resistance to *F. graminearum* was also observed in the *sid2* mutant (Fig. 2d,e). These results suggest that the enhanced disease resistance caused by NMN is also regulated by an SA-independent signalling pathway. As stated above, NMN-derived antifungal metabolites may be involved in disease resistance to *F. graminearum*,

Furthermore, we performed a transcriptome analysis using an Agilent Arabidopsis 3 44k Microarray (Palo Alto, CA, USA) to profile NMN-induced disease resistance. We identified 196 up-regulated and 92 down-regulated genes (Fold Change > 2, P < 0.05) in NMN-treated leaves compared to water-treated leaves without inoculation. In addition, we also identified 688 up-regulated and 786 down-regulated genes (Fold Change > 2, P < 0.05) in NMN-pretreated leaves inoculated with *F. graminearum* compared to water-pretreated leaves inoculated with *F. graminearum*. We then performed a gene ontology (GO) enrichment analysis. Table S1 shows the top 30 GO terms enriched in genes induced by NMN without inoculation. These GO terms include response of SA stimulus, SAR, immune response, and negative regulation of cell death. These genes were up-regulated prior to inoculation with *F. graminearum*. These data support the hypothesis that SA signalling pathway activation and cell death repression are involved in NMN-induced disease resistance against *F. graminearum* (Fig. 3). Table S2 shows the top 30 GO terms enriched in genes induced in plants that were pretreated with NMN and collected 3 days post inoculation (dpi) with *F. graminearum*. Table S2 suggests that SA-dependent signalling, the immune response, and systemic acquired resistance were also activated by NMN pretreatment in leaves inoculated with *F. graminearum*. The number of genes in each GO term was apparently increased compared with those in Table S1. Table S3 suggests that both the JA and ET signalling pathways are suppressed by NMN pretreatment in inoculated leaves. These data also indicate that suppression of JA and ET signalling contributed to NMN-induced disease resistance against *F. graminearum*. Table S3 also suggests that the ABA signalling pathway and abiotic stress response were repressed by NMN pretreatment. Thus, NMN pretreatment may optimize the defence signalling pathways, leading to enhanced resistance to *F. graminearum*.

A hierarchical clustering analysis of NMN-regulated genes containing SA-, JA-, and ET-related GO terms was performed (Fig. 3). NMN-induced SA-related genes (Fig. 3a) in *F. graminearum*-inoculated leaves included *cysteine-rich receptor-like protein kinase 45* (*CRK45*)^27^, *ACCELERATED CELL DEATH 6* (*ACD6*)^28^, *WRKYs* ^29^, *calcium-dependent protein kinase 31* (*CPK31*)^30^, and *calmodulin-binding protein 25* (*CAMBP25*)^31^. The *CRK45* and *WRKY53* genes have been reported to positively regulate disease resistance against *Pst*DC3000^27, 32^. In addition, *ACD6* is required for SA-dependent disease resistance against virulent bacterial pathogens^28^. These results suggest that these genes are also involved in disease resistance to *F. graminearum*. Since calcium signalling is also known to be involved in the defence signalling activated by extracellular pyridine nucleotides^13^, the *CPK31* and *CAMBP25* genes may function in NMN-induced disease resistance^30, 31^. Interestingly, the expression of *azelaic acid induced 1* (*AZI1*), which is involved in the systemic immunity triggered by pathogen infection and azelaic acid^26, 33^, was up-regulated by NMN treatment with or without inoculation. In addition to the *ICS1* gene, expression of the *UGT74F1* gene, which catalyses the formation of SA-glucoside (SAG) and the glucose ester of SA (SGE), was significantly reduced by NMN treatment in inoculated leaves^34^. The down-regulation of the *UGT74F1* gene likely contributed to the accumulation of free SA in NMN-treated leaves inoculated with *F. graminearum*. The *DOX1* gene, which is involved in protection against oxidative stress, is reportedly up-regulated by high NAD content^25^. Similarly, the expression of the *DOX1* gene was up-regulated by NMN treatment in uninoculated leaves. However, the induction of *DOX1* expression in response to *F. graminearum* inoculation was down-regulated by pretreatment with NMN. We examined hydrogen peroxide accumulation in water- or NMN-pretreated leaves inoculated with *F. graminearum* by DAB staining (Fig. S5). These results indicated that NMN pretreatment suppressed the ROS (reactive oxygen species) generation induced by *F. graminearum* inoculation in Arabidopsis leaves. The suppression of ROS accumulation by NMN was likely involved in the down-regulation of the *DOX1* gene by NMN in infected leaves. Therefore, ROS accumulation may negatively affect disease resistance against *F. graminearum* in Arabidopsis leaves, since host cell death caused by ROS likely contributes to the virulence of *F. graminearum* necrotrophic growth. Figure 3b shows that JA-inducible (JAZs and VSP1) and JA-biosynthetic genes (DGL and AOC2) were down-regulated by NMN treatment in inoculated leaves^35, 36^. Although the expression of the *WRKY50* and *WRKY51* genes was induced by NMN pretreatment in inoculated leaves, these genes control the repression of JA-dependent signalling in low oleic acid (18:1)-containing Arabidopsis plants^37^. These data indicated that the JA-dependent signalling pathway was suppressed by NMN pretreatment in inoculated leaves, resulting in enhanced disease resistance against *F. graminearum* ^6^.

For the ET-related genes, the expression of 7 *ERFs* (ethylene-responsive transcription factors) was down-regulated by NMN pretreatment in inoculated leaves (Fig. 3c). Five of these genes belong to the ERF subfamily^38, 39^. In particular, the expression of *ERF-1* and *ERF96* was induced by ET treatment and activated the transcription of the *PDF1.2a* gene through a GCC box^40, 41^. Therefore, the down-regulation of these genes is likely involved in the suppression of the ET response in leaves inoculated with *F. graminearum*. Figure 3c also shows 7 *ERFs* (lower parts of the cluster) that are induced by NMN after *F. graminearum* inoculation. These genes belong to the DREB subfamily^38, 39^. Among them, *RAP2.1* and *DEAR2* have EAR repression domains and act as transcriptional repressors^42, 43^. Thus, suppression of ET-dependent signalling by NMN pretreatment in inoculated leaves likely contributes to enhanced disease resistance against *F. graminearum* ^26^.

We next quantified the amount of NMN and NA in NMN-treated Arabidopsis leaves with or without *F. graminearum* inoculation (Fig. S6). NMN and NA were apparently increased 6 h after NMN spraying (Fig. S6b). In the inoculated leaves, NMN and NA accumulation was also enhanced by NMN pretreatment (Fig. S6c).

Furthermore, we quantified both NAD(H) and NADP(H) levels in leaves with and without NMN treatment (Fig. S7). Extracellular NMN induced NAD and NADP accumulation after 6 h (0 h of inoculation). By contrast, the contents of the reduced forms NADH and NADPH were not significantly altered by NMN treatment. The contents of NAD and NADP were also increased by the inoculation of *F. graminearum* in Arabidopsis leaves (Fig. S7). Therefore, changes in the NAD+/NADH balance may affect defence signalling. Further increases in the NAD and NADP content were observed in inoculated leaves pretreated with NMN (Fig. S7). These results suggested that extracellular NMN induced disease resistance against *F. graminearum* without inducing host cell death.

We then investigated the effects of NMN pretreatment and *F. graminearum* inoculation on the expression of the representative NAD biosynthetic genes *nicotinamide mononucleotide adenyltransferase* (*NMNAT*)^44^ and *nicotinamidase 2* (*NIC2*) (Fig. S8). The expression of both genes was induced by *F. graminearum* inoculation but not by NMN treatment. Since NMNAT is a rate-limiting enzyme for NAD biosynthesis, the induction of NMNAT likely contributes to the accumulation of NAD. In addition, the induction of the *NIC2* gene may be involved in the biosynthesis of pyrimidine alkaloids such as trigonellin^13^ (Fig. S6a, S8b).

**Figure 4:**
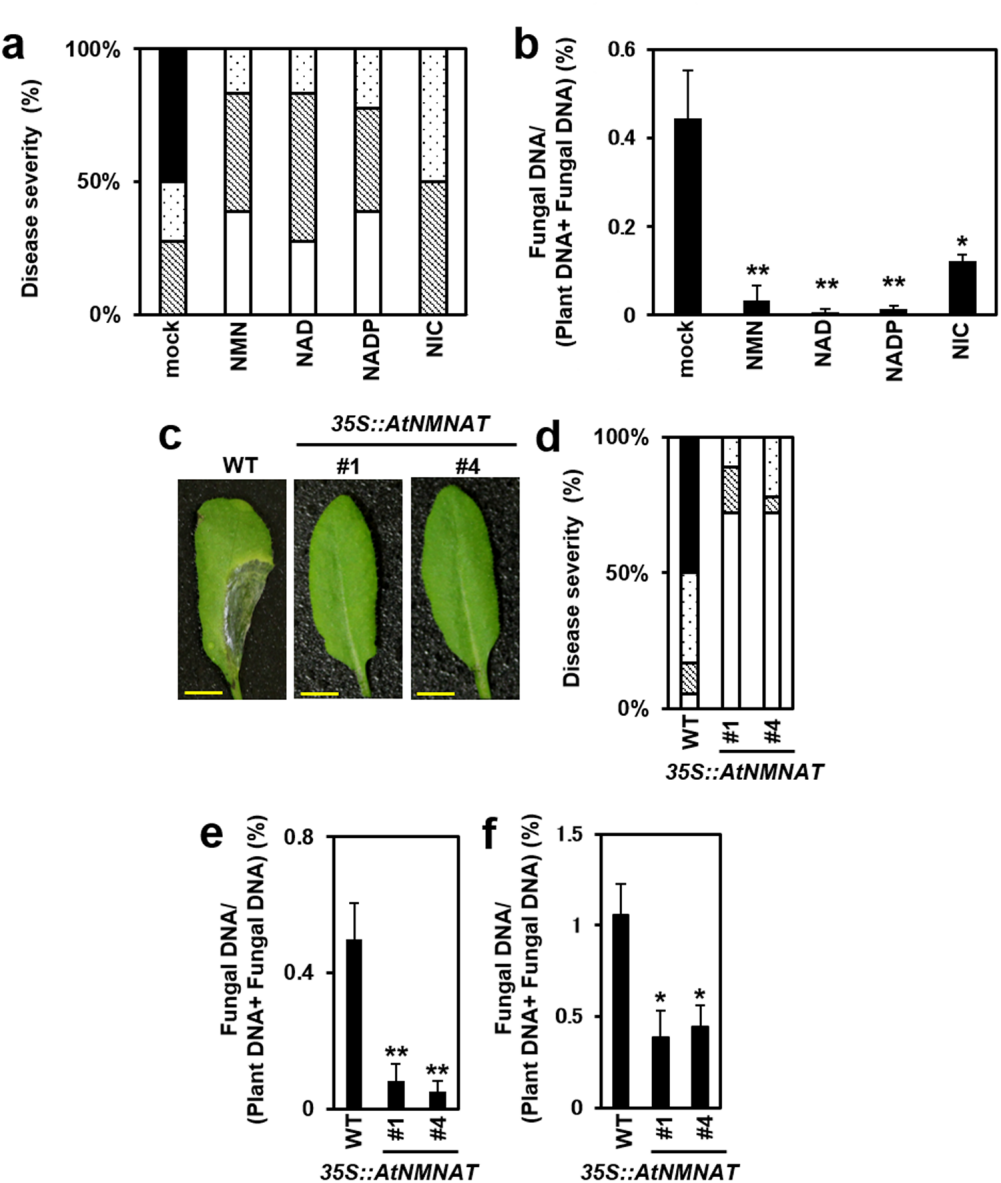
The effects of pretreatment with pyridine nucleotides and overexpression of the *NMNAT* gene on disease resistance against *F. graminearum* in Arabidopsis. Water (mock), NMN, NAD, NADP and NIC (0.3 mM) were sprayed onto the surface of Arabidopsis rosette leaves prior to incubation for 6 h. A conidia solution (1 × 10^5^ conidia/ml) of *F. graminearum* was then injected into leaves. (**a**) The disease severity was evaluated by the visible symptoms of inoculated leaves 3 days after inoculation (n = 18). Open box: normal, cross-hatched box: colour change, dot box: partial aerial mycelium, closed box: expanded aerial mycelium. (**b**) Pretreatment with pyridine nucleotides was carried out as described above. *F. graminearum* gDNA was measured by qPCR. Error bars represent the standard deviation (n = 3) (Student’s t-test *P < 0.05 **P < 0.01). (**c**) Transgenic plants (*35 S::AtNMNAT*) show enhanced disease resistance. Conidia solutions (1 × 10^5^ conidia/ml) of *F. graminearum* were inoculated into transgenic plant (*35 S::AtNMNAT*) leaves and flower buds. Photographs of representative leaves from WT and transgenic plants (*35 S::AtNMNAT*) at 3 days after inoculation. Scale bars: 1 cm. (**d**) The disease severity was evaluated by the disease symptoms of inoculated leaves 3 days after inoculation (n = 18). Open box: normal, cross-hatched box: colour change, dot box: partial aerial mycelium, closed box: expanded aerial mycelium. (**e**) *F. graminearum* gDNA was measured by qPCR in inoculated leaves. Error bars represent the standard deviation (n = 3) (Student’s t-test **P < 0.01). (**f**) *F. graminearum* gDNA was measured by qPCR in inoculated flower buds. Error bars represent the standard deviation (n = 3) (Student’s t-test *P < 0.05).

As stated above, NMN is a precursor of NAD, a coenzyme and essential redox-active constituent of all living organisms^21^. We examined whether other pyridine nucleotides in the NAD biosynthesis pathway also affect disease resistance against *F. graminearum*. Pretreating plants with NAD and NADP by spraying significantly decreased disease symptoms in Arabidopsis leaves compared with water treatment (Fig. 4a,b). Quantification of gDNA derived from *F. graminearum* also indicated enhanced disease resistance with NAD and NADP treatment (Fig. 4b). These effects were comparable to those of NMN. On the other hand, NIC had a relatively weak effect on disease resistance compared with the other metabolites. Taken together, these findings suggest that NMN, NAD and NADP are effective suppressors of *F. graminearum*-induced disease development in Arabidopsis.

Furthermore, we prepared Arabidopsis transgenic plants to examine the involvement of intracellular NAD biosynthesis in disease resistance against *F. graminearum*. Based on the effects of NAD-related metabolites on *Fusarium* resistance, we prepared transgenic plants constitutively expressing NMNAT, which catalyses the conversion of NMN to NAD (salvage pathway) and NaMN to NaAD (common pathway; Fig. S6a ^44^). NMNAT is a rate-limiting enzyme involved in NAD biosynthesis in plants^41^. In addition, expression of the *NMNAT* gene was induced by *F*. *graminearum* inoculation, suggesting that NMNAT plays a role in defence signalling in response to *F*. *graminearum* (Fig. S8a). NAD contents were increased in both inoculated and uninoculated leaves of two independent *35S*::*AtNMNAT* lines compared with those of WT plants, whereas the NADP content was unchanged (Fig. S7). By contrast, NMN pretreatment increased the metabolic pool of the NAD biosynthetic pathway (Fig. S6, S7). These transgenic plants showed weak disease symptoms following inoculation with *F. graminearum* compared to the WT plants (Fig. 4c–g). In addition, the amount of *F. graminearum* genomic DNA in these transgenic plants was also significantly decreased compared with the WT plant (Fig. 4e). Quantification of gDNA derived from *F. graminearum* also indicated enhanced disease resistance in flowers of the transgenic plants (Fig. 4f). We found that the level of *PR1* mRNA increased in transgenic plants without inoculation compared with WT plants (Fig. S9a). However, enhanced accumulation of *PR1* mRNA was not observed in the inoculated leaves of *35S::AtNMNAT* plants. The constitutive activation of the SA-dependent signalling pathway is likely involved in the enhanced disease resistance to *F. graminearum* in *35S::AtNMNAT* plants. *PDF1.2a* expression was suppressed in *F. graminearum*-inoculated transgenic plants (Fig. S9b). Thus, the higher NAD content in the *35S::AtNMNAT* plants resulted in enhanced disease resistance against *F. graminearum* through the activation of SA signalling and the suppression of JA/ET signalling.

### NMN pretreatment also activates disease resistance against *F. graminearum* in barley plants

**Figure 5:**
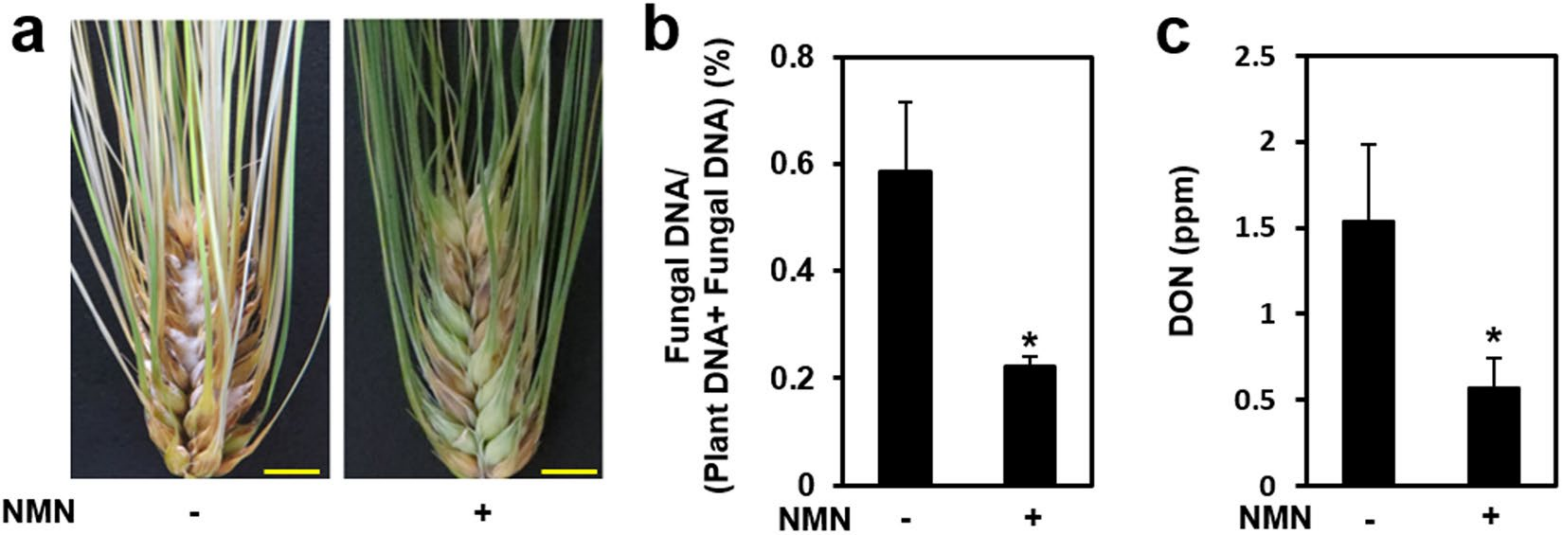
NMN pretreatment induces disease resistance against *F. graminearum* in barley. (**a**) NMN was sprayed on spikes of the FHB-susceptible line H.E.S.4, followed by incubation for 4 h. A conidia solution (1 × 10^4^ conidia/ml) of *F. graminearum* was then applied by spraying (n = 3). (**a**) Scale bars: 1 cm. (**b**) *F. graminearum* gDNA was measured by qPCR. Error bars represent the standard deviation (n = 3) (Student’s t-test *P < 0.05). (**c**) DON was measured by QuickScan DON3 (n = 3) (Student’s t-test *P < 0.05).

Since NMN and related metabolites activated disease resistance against *F. graminearum* in Arabidopsis leaves and flowers, we also investigated whether it could do so in the FHB-susceptible barley cultivar H.E.S.4, which shows very weak resistance to *F. graminearum*. Disease symptoms such as aerial hyphae were observed in the water-pretreated flowers (spikes) of H.E.S.4, whereas NMN pretreatment significantly alleviated the disease symptoms (Fig. 5a). In addition, genomic DNA of *F. graminearum* in NMN-pretreated spikes was significantly decreased compared with plants that received the mock pretreatment (Fig. 5b). DON accumulation in NMN-pretreated spikes was also decreased compared with water-pretreated spikes (Fig. 5c). These results indicate that NMN is also effective in controlling *F. graminearum*-induced disease injury and mycotoxin accumulation in barley.

In this study, we found that NMN, which is a precursor of NAD, is enriched in FHB-resistant barley cultivars compared with susceptible ones. Although NMN does not have antifungal activity, exogenous application of NMN enhanced disease resistance to *F. graminearum* in Arabidopsis leaves and flowers. This NMN-induced *Fusarium* resistance is controlled by the activation of the SA-mediated signalling pathway and repression of the JA/ET-dependent signalling pathway in Arabidopsis. NMN pretreatment induced the expression of the *ICS1* gene and resulted in increased SA content in Arabidopsis leaves inoculated with *F. graminearum*. This enhanced SA accumulation likely suppressed JA biosynthesis and JA/ET responses in Arabidopsis leaves. A higher NAD content in plants is sufficient to enhance disease resistance against *F. graminearum* through the activation of SA signalling and suppression of JA/ET signalling. However, enhanced resistance was also observed in the SA-deficient *sid2* mutant (Fig. 2e). In addition, the increase in SA accumulation mediated by NMN in *F. graminearum*-infected leaves was less than 2-fold (Fig. 2c). Therefore, an SA-independent signalling pathway is also involved in NMN-induced disease resistance. Thus, elucidating both SA-dependent and SA-independent signalling pathways is necessary to understand the effects of NMN and related metabolites on disease resistance against *F. graminearum*. How do NAD and related metabolites activate the defence signalling pathways leading to enhanced resistance against *F. graminearum*? As stated above, an imbalance in the NAD+/NADH and NADP/NADPH ratios affects not only various NAD- and NADP-dependent enzymatic reactions but also defence signalling pathways^13^. NAD-dependent protein deacetylases (SIRTs) are activated by an increased NAD+/NADH ratio^14^. Furthermore, target proteins such as histones are deacetylated and then poly(ADP-ribosyl)ated by SIRT and PARP (poly(ADP-ribose)polymerase), resulting in the modulation of the expression of many genes^14^. Since SIRTs and PARPs are also found in plants, similar events may trigger transcriptional activation of defence-related genes such as *ICS1*. Finally, we revealed that NMN pretreatment induced disease resistance against *F. graminearum* in an FHB-susceptible barley cultivar (H.E.S.4). Thus, modifying the content of NAD-related metabolites is useful for the control of disease injury and mycotoxin accumulation in plants.

## Methods

### Plant materials and growth conditions

*Arabidopsis thaliana* (ecotype Columbia; Col-0) and its derivatives were used in this study. The *salicylic acid induction-deficient 2-2* (*sid2-2*) mutant^45^ was obtained from the Arabidopsis Biological Resource Center (ABRC). *35S::AtNMNAT* (At5g55810) transgenic plants were prepared in this study. Arabidopsis seeds were sown in soil at 4 °C in the dark for 2 days and thereafter grown at 22 °C under a 16/8-h light/dark cycle. The FHB-susceptible cultivars Turkey 45 (2-row) and H.E.S.4 (6-row) and FHB-resistant cultivars Maja and Sirius O-525 were grown in the field of the Institute of Plant Science and Resources, Okayama University, as previously described^19^.

### Fungal material and growth conditions

*F. graminearum* strain H3 was used in this study^46^. The fungus was grown at 28 °C on potato dextrose agar (PDA; Nippon Becton Dickinson Company, Ltd., Tokyo, Japan) medium. *F. graminearum* conidia were prepared using SN liquid medium, which is composed of 0.1% KH_2_ PO_4_, 0.1% KNO_3_, 0.1% MgSO_4_·7H_2_O, 0.05% KCl, 0.02% glucose, and 0.02% sucrose^47^.

### Fusarium head blight (FHB) assay (cut-spike test)

The cut-spike test using field-grown barley spikes was performed as previously described^19^. Spikes were sprayed with an NMN solution with 0.001% silwetL77. After 4 h, an *F. graminearum* conidia solution (1 × 10^4^ conidia/ml) with 0.001% silwetL77 was sprayed on the spikes. Inoculated spikes were collected at 7 dpi for disease incidence determination (colour change of barley grains), *F. graminearum* gDNA analysis and quantification of DON.

### Pyridine nucleotide treatment

Pyridine nucleotides (NMN, NAD, NADP and NIC; Sigma-Aldrich Japan, Tokyo, Japan) were dissolved in water at a concentration of 300 µM, and the pH was adjusted to 6.0 with 1 N NaOH. The resulting solution plus 0.001% silwetL-77 was sprayed onto Arabidopsis leaves, flowers and barley spikes (H.E.S.4). To analyse disease resistance, sprayed leaves and flowers were inoculated with *F. graminearum* 6 h after treatment. Sprayed barley spikes were inoculated with *F. graminearum* 4 h after treatment.

### Antifungal activity assay

Conidial suspensions of *F. graminearum* (1 × 10^3^ conidia/ml) were grown in SN liquid medium for 2 days with or without NMN. The growth of *Fusarium* was measured by a 3-(4,5-di-methylthiazol-2-yl)-2,5-diphenyltetrazollium bromide (MTT) assay^2^.

### *F. graminearum* infection

To prepare conidia solutions, *F. graminearum* was cultured in SN liquid medium at 22 °C for 3 days. The conidia were then collected by centrifugation and washed with phosphate-buffered saline (PBS) at least 3 times. The collected conidia were suspended in PBS, and the number of conidia was counted using a haemocytometer^2^. Arabidopsis leaves were sprayed with NMN, NAD, NADP, or NIC plus 0.001% silwetL77. After 6 h, conidia solutions were injected into Arabidopsis leaves. For infiltration inoculation, a conidial suspension of *F. graminearum* (1 × 10^5^ conidia/ml) was injected into the abaxial side of the leaves using a needleless syringe^2^. Inoculated plants were incubated in a chamber under approximately 100% relative humidity at 22 °C for 3 days. Disease development on leaves was assessed as described previously^2^. Inoculated leaves were collected at 3 dpi to for further analysis.

Flowering plants were used for the inoculation assay. Flowers were sprayed with an NMN solution with 0.001% silwetL77. After 6 h, *F. graminearum* conidia solutions (1 × 10^5^ conidia/ml) with 0.001% silwetL77 were sprayed on Arabidopsis flowers as previously described^47^. The inoculated plants were then kept in a dew chamber at 100% relative humidity for 7 days. Inoculated flowers were collected at 7 dpi for further analysis.

### DAB staining

To detect H_2_O_2_, inoculated leaves were infiltrated under a gentle vacuum with 1 mg ml^−1^ DAB containing 0.05% v/v Tween 20 and 10 mM PBS at pH 7.0. The leaves were then fixed by boiling for 15 min in ethanol: acetic acid: glycerol at 3:1:1 and were then observed under a microscope^48^. We used an OLYMPUS BX-50 microscope (Olympus Optical Co., Ltd., Tokyo, Japan).

### DNA isolation and quantification of *F. graminearum* gDNA

Genomic DNA was isolated using a Nucleon Phytopure Kit (GE Healthcare., Tokyo, Japan). The amount of *F. graminearum g*DNA in inoculated leaves and flowers was quantified by qPCR. For inoculated flower samples, primary inflorescences of approximately 5 cm were cut from approximately 6-week-old Arabidopsis plants. qPCR was performed an Mx3000P instrument (Stratagene Japan Inc., Tokyo, Japan) with 2X SYBR Premix Ex Taq II (Takara Bio Inc., Shiga, Japan). The Arabidopsis *Act2/8* gene, barley *GAPDH* gene and *Fusarium EF1α* gene were used for analysis. *ACT2/8* was amplified using the primers *Act2/8_*F-1, GGTAACATTGTGCTCAGTGGTGG and *Act2/8_*R-1, AACGACCTTAATCTTCATGCTGA, and the *EF1α* gene was amplified using the primers *EF1α_*F-1, CCATTCCCTGGGCGCT and *EF1α_*R-1, CCTATTGACAGGTGGTTAGTGACTGG. *HvGAPDH* was amplified using the primers *HvGAPDH_*F-1, TTGACAGGAACCCTGAGGAG and *HvGAPDH_*R-1, TGTGAATGAGCGAAAACCAG. qPCR analysis was performed using an Mx3000P instrument (Stratagene Japan Inc., Tokyo, Japan). To generate a standard curve, homologous standards were used in all experiments. The initial gDNA quantities were calculated by the standard curve method using MxPro software (Agilent).

### Measurements of NAD/NADH and NADP/NADPH

Samples were ground to fine powder in liquid nitrogen. Then, 40 mg of each sample was used for NAD/NADH and NADP/NADPH quantification. NAD/NADH and NADP/NADPH accumulation was measured using a Fluorescent NAD/NADH and NADP/NADPH Detection Kit (Cell Technology Inc., Mountain View, CA) according to the manufacturer’s protocol.

### RT-qPCR

Total RNA was isolated using a Plant RNA Isolation Mini Kit (Agilent Technologies., CA, USA). First-strand cDNA was synthesized using the PrimeScript RT Reagent Kit (Takara Bio Inc., Shiga, Japan). RT-qPCR (intercalator method) was performed as described above. The following primers were used for RT-qPCR: *PR1* ^49^, *PDF1.2a* ^46^. *ICS1_*F-1, ATGAGATTCAGCCTCGCTGT and *ICS1_*R-1, TGATGGATCTCCAATCGTCA. *NMNAT*_F-1, CCAACGAACTTTGACGGTTT and *NMNAT_*R-1, CGGGAGTGCAGAAAGATAGC. *NIC2_*F-1, CGAGAACGTACGAGACACGA and *NIC2_*R-1, TTCCGTCGAGGATTAGATCG. The primers used to amplify the *Act2/8* gene are listed above. To generate a standard curve, homologous standards were used in all experiments. The cDNA quantities of target genes were calculated by the standard curve method using MxPro software (Agilent), and all quantifications in Arabidopsis were normalized to the mRNA level of the *Actin2/8* reference gene.

### Generation of transgenic plants

To create *35S::AtNMNAT* Arabidopsis transgenic plants, Arabidopsis *NMNAT* (At5g55810) cDNA was amplified using the primers *AtNMNAT*_F-1, CACCATGGATGTCCCGTTACCAGT and *AtNMNAT*_R-1, TCATGTGAGCTCAGTGTATAGTTGAT, and recombined into the Gateway^TM^ vector pENTR^TM^/D-TOPO (Invitrogen., Carlsbad., CA, USA). The gene was then sequenced and transferred into the transformation vector pK2GW7.0^50^. This plasmid was then transformed into *Agrobacterium tumefaciens* strain GV2260. Transgenic Arabidopsis plants were generated according to the *Agrobacterium*-mediated floral dip method^51^. Transgenic plants were selected on Murashige and Skoog (MS) medium supplemented with 2% sucrose and 0.8% Bacto agar at pH 5.8 and with 50 µg ml^−1^ kanamycin.

### Quantification of DON

Barley spikes were ground to fine powder in liquid nitrogen. Then, 200 mg of each sample was used for DON quantification. DON accumulation was measured using a QuickTox^TM^ Kit for QuickScan DON3 (ENVIRLogix, Inc., Portland, Maine, USA) according to the manufacturer’s protocol.

### Microarray analysis

Total RNA was prepared from 8 samples (2 biological replicates for 4 different treatments) using a Plant RNA Isolation Mini Kit (Agilent Technologies., CA, USA). Four different treatments were analysed: (1) water-treated Arabidopsis leaves, (2) NMN-treated leaves, (3) water-treated, *F. graminearum*-inoculated leaves, and (4) NMN-treated, *F. graminearum*-inoculated leaves. For (1) and (2), these leaves ware collected at 6 h after the spraying of water or NMN. For (3) and (4), after water or NMN pretreatment, *F. graminearum*-inoculated leaves were collected at 3 dpi. RNA quality was assessed using the 2200 TapeStation (Agilent Technologies), and a one-colour microarray experiment was performed using the Agilent Arabidopsis ver. 3 oligomicroarray, according to the manufacturer’s 60-mer oligo microarray processing protocol. Total RNAs (200 ng) were used to prepare Cy3-labeled cRNA, using a Low Input Quick Amp Labeling Kit (Agilent Technologies). The hybridized and washed arrays were scanned at maximum laser intensity in the Cy3 channel using an Agilent microarray scanner (G2565BA; Agilent Technologies). The images were analysed using Feature Extraction Software (Ver. 10.7.3.1; Agilent Technologies). These data were analysed further with GeneSpring GX12.5 software (Agilent Technologies). Normalization was performed as follows: 1, intensity-dependent Lowess normalization; 2, data transformation, with measurements less than 0.01 set to 0.01; 3, per-chip normalization, in which the 75th percentile method was used to normalize each array; and 4, per-gene normalization, in which the data were normalized to the median value of 8 measurements. After normalization, statistically significant gene sets were defined as those exhibiting P values below the cut-off of 0.05. Genes with a fold change (FC) in the expression ratio (water-treated vs NMN-treated) >2, either up-regulated or down-regulated, were identified. Thus, a combination of statistical analyses and FC methods was used. In addition, we calculated false discovery rate (FDR) by Benjamini-Hochberg procedure. GO enrichment analyses were also performed using GeneSpring GX12.5. Using Fisher’s exact test (corrected P < 0.01), we identified the top 30 GO categories significantly enriched in genes that were up-regulated by NMN treatment. For the hierarchical clustering, SA-, JA- and ET-related genes were selected from the NMN-induced or NMN-suppressed genes (FC > 2, P < 0.05) with or without inoculation, based on their GO term names. For the clustering analysis, we eliminated genes that over-lapped among these categories. Then, hierarchical clustering analysis was performed using GeneSpring GX ver.12.5. The microarray data from this publication have been submitted to the GEO database (http://www.ncbi.nlm.nih.gov/geo/) and assigned the identifier GSE93079.

### Metabolome analysis

The freeze-dried samples were measured and added to extraction buffer (0.1% (v/v) formic acid in 80% (v/v) MeOH) containing internal standards (positive: 33.6 nM lidocaine, negative: 210 nM 10-camphorsulfonic acid). The extracted solutions of all samples were adjusted to the same concentration (4 mg dry weight/mL). Then, they were diluted 10 times with extraction buffer, and 25 µL of each sample was dried with N_2_ gas. The dried samples were dissolved in 250 µL of LC-MS grade H_2_O. After filtration (UNIFILTER 384 well, Whatman), 1 µL of the solution was measured by LC-QqQ-MS^52–54^. Then, the peak area of each metabolite was normalized to the peak area of the internal standard. Using the resulting MS intensity of each metabolite per dried weight, we compared each treatment. The differentially accumulated metabolites were identified by statistical analysis.

## Electronic supplementary material

Supplementary Information
