# Supplementary material for: Nicotinamide mononucleotide and related metabolites induce disease resistance against fungal phytopathogens in Arabidopsis and barley
Source: Sci Rep. 2017 Jul 25;7:6389. doi: 10.1038/s41598-017-06048-8 (PMC5526872; doi:10.1038/s41598-017-06048-8)
Supplement: Supplementary file 1 — Supplementary Information [file 41598_2017_6048_MOESM1_ESM.pdf]

Supplementary information: 3 supplementary tables and 9 supplementary figures.

Title: Nicotinamide mononucleotide and related metabolites induce disease resistance against fungal phytopathogen in Arabidopsis and barley.

Authors: Akihiro Miwa, Yuji Sawada, Daisuke Tamaoki, Masami Hirai, Makoto Kimura, Kazuhiro Sato & Takumi Nishiuchi

Supplementary Table 1. Top 30 enriched GO categories of NMN-induced genes without inoculation (NMN-pretreated vs water-pretreated leaves without inoculation of *F. graminearum*)

GO accession	GO term	Count in Selection	% Count in Selection	Count in Total	% Count in Total	Corrected p-value[a]
GO:0009751	response to salicylic acid stimulus	19	11.4	475	1.92	2.3E-06
GO:0006865|GO:0006866	amino acid transport	15	9.0	274	1.10	2.3E-06
GO:0046942	carboxylic acid transport	15	9.0	297	1.20	4.7E-06
GO:0015849	organic acid transport	15	9.0	303	1.22	4.7E-06
GO:0050832|GO:0042831	defense response to fungus	14	8.4	343	1.38	1.5E-04
GO:0071446	cellular response to salicylic acid stimulus	13	7.8	352	1.42	9.5E-04
GO:0009863	salicylic acid mediated signaling pathway	13	7.8	350	1.41	9.5E-04
GO:0009862	systemic acquired resistance, salicylic acid mediated signaling pathway	11	6.6	251	1.01	1.2E-03
GO:0009620|GO:0009621	response to fungus	15	9.0	495	2.00	1.2E-03
GO:0009581	detection of external stimulus	6	3.6	61	0.25	2.9E-03
GO:0010310	regulation of hydrogen peroxide metabolic process	9	5.4	187	0.75	3.9E-03
GO:0009595|GO:0009596	detection of biotic stimulus	7	4.2	105	0.42	5.0E-03
GO:0009627	systemic acquired resistance	13	7.8	438	1.77	5.6E-03
GO:2000377	regulation of reactive oxygen species metabolic process	9	5.4	203	0.82	5.9E-03
GO:0009814	defense response, incompatible interaction	14	8.4	528	2.13	7.7E-03
GO:0071702	organic substance transport	15	9.0	612	2.47	8.2E-03
GO:0043069|GO:0043072	negative regulation of programmed cell death	8	4.8	169	0.68	8.2E-03
GO:0045087|GO:0002226	innate immune response	16	9.6	692	2.79	8.2E-03
GO:0009607	response to biotic stimulus	24	14.5	1399	5.64	8.2E-03
GO:0006955	immune response	16	9.6	697	2.81	8.2E-03
GO:0006952|GO:0002217|GO:0042829	defense response	24	14.5	1397	5.63	8.2E-03
GO:0051707|GO:0009613|GO:0042828	response to other organism	24	14.5	1398	5.64	8.2E-03
GO:0060548	negative regulation of cell death	8	4.8	173	0.70	8.4E-03
GO:0045088	regulation of innate immune response	12	7.2	418	1.69	9.4E-03
GO:0050776	regulation of immune response	12	7.2	420	1.69	9.5E-03
GO:0002682	regulation of immune system process	12	7.2	423	1.71	9.8E-03
GO:0010363	regulation of plant-type hypersensitive response	11	6.6	371	1.50	1.3E-02
GO:0002376	immune system process	17	10.2	828	3.34	1.3E-02
GO:0006612	protein targeting to membrane	11	6.6	378	1.52	1.5E-02
GO:0080135	regulation of cellular response to stress	11	6.6	384	1.55	1.7E-02

[a]Each GO category was significantly enrichment in the NMN-regulated gene by a Fisher's exact test (corrected p value).

Supplementary Table 2. Top 30 enriched GO categories of NMN-induced genes after inoculation of *F. graminearum*.
(NMN-pretreated vs water-pretreated leaves 3 days after inoculation of *F. graminearum*)

GO ACCESSION	GO Term	Count in Selection	% Count in Selection	Count in Total	% Count in Total	Corrected p-value[a]
GO:0006952|GO:0002217|GO:0042829	defense response	94	15.6	1397	5.63	2.9E-15
GO:0002376	immune system process	62	10.3	828	3.34	3.1E-11
GO:0009627	systemic acquired resistance	43	7.1	438	1.77	4.5E-11
GO:0009607	response to biotic stimulus	82	13.6	1399	5.64	3.6E-10
GO:0051707|GO:0009613|GO:0042828	response to other organism	82	13.6	1398	5.64	3.6E-10
GO:0009814	defense response, incompatible interaction	45	7.5	528	2.13	8.6E-10
GO:0010167	response to nitrate	26	4.3	196	0.79	4.9E-09
GO:0045087|GO:0002226	innate immune response	50	8.3	692	2.79	1.5E-08
GO:0015706|GO:0006872	nitrate transport	26	4.3	210	0.85	1.7E-08
GO:0006955	immune response	50	8.3	697	2.81	1.7E-08
GO:0009696	salicylic acid metabolic process	26	4.3	216	0.87	2.5E-08
GO:0015698	inorganic anion transport	30	5.0	286	1.15	2.5E-08
GO:0006820|GO:0006822	anion transport	34	5.6	362	1.46	2.5E-08
GO:0009697	salicylic acid biosynthetic process	25	4.1	208	0.84	5.7E-08
GO:2000377	regulation of reactive oxygen species metabolic process	24	4.0	203	0.82	1.8E-07
GO:0010310	regulation of hydrogen peroxide metabolic process	23	3.8	187	0.75	1.9E-07
GO:0051704|GO:0051706	multi-organism process	86	14.3	1741	7.02	2.1E-07
GO:0009862	systemic acquired resistance, salicylic acid mediated signaling pathway	26	4.3	251	1.01	5.0E-07
GO:0000041	transition metal ion transport	24	4.0	244	0.98	6.1E-06
GO:0009863	salicylic acid mediated signaling pathway	29	4.8	350	1.41	8.3E-06
GO:0071446	cellular response to salicylic acid stimulus	29	4.8	352	1.42	8.9E-06
GO:0010106	cellular response to iron ion starvation	16	2.7	116	0.47	1.4E-05
GO:0009617|GO:0009618|GO:0009680	response to bacterium	38	6.3	568	2.29	1.4E-05
GO:0009751	response to salicylic acid stimulus	34	5.6	475	1.92	1.5E-05
GO:0006950	response to stress	140	23.2	3717	14.99	2.6E-05
GO:0006857	oligopeptide transport	15	2.5	110	0.44	4.0E-05
GO:0006826|GO:0015681	iron ion transport	16	2.7	126	0.51	4.0E-05
GO:0031348	negative regulation of defense response	24	4.0	275	1.11	4.1E-05
GO:0015833	peptide transport	15	2.5	112	0.45	4.7E-05
GO:0050832|GO:0042831	defense response to fungus	27	4.5	343	1.38	5.1E-05

aEach GO category was significantly enrichment in the NMN-regulated gene by a Fisher's exact test (corrected P-value).

Supplementary Table 3. Top 30 enriched GO categories of NMN-suppressed genes after inoculation of *F. graminearum*. (NMN-pretreated vs water-pretreated leaves 3 days after inoculation of *F. graminearum*).

GO accession	GO term	Count in selection	% Count in selection	Count in total	% Count in total	Corrected p-value[a]
GO:0009723	response to ethylene stimulus	50	7.3	346	1.40	1.04E-17
GO:0042221	response to chemical stimulus	189	27.7	3479	14.03	2.01E-17
GO:0010033	response to organic substance	152	22.3	2665	10.75	5.02E-15
GO:0014070	response to organic cyclic compound	27	4.0	125	0.50	2.25E-13
GO:0010583	response to cyclopentenone	27	4.0	125	0.50	2.25E-13
GO:0006950	response to stress	183	26.8	3717	14.99	1.14E-12
GO:0009611|GO:0002245	response to wounding	42	6.2	341	1.38	1.15E-12
GO:0009719	response to endogenous stimulus	101	14.8	1561	6.30	1.33E-12
GO:0009725	response to hormone stimulus	101	14.8	1559	6.29	1.33E-12
GO:0050896|GO:0051869	response to stimulus	264	38.7	6202	25.01	1.52E-12
GO:0009694	jasmonic acid metabolic process	28	4.1	155	0.63	3.64E-12
GO:0009407	toxin catabolic process	32	4.7	210	0.85	4.93E-12
GO:0009404	toxin metabolic process	32	4.7	210	0.85	4.93E-12
GO:0031407	oxylipin metabolic process	28	4.1	162	0.65	9.26E-12
GO:0009753	response to jasmonic acid stimulus	48	7.0	474	1.91	9.71E-12
GO:0009695	jasmonic acid biosynthetic process	24	3.5	132	0.53	2.16E-10
GO:0031408	oxylipin biosynthetic process	24	3.5	138	0.56	5.62E-10
GO:0009414	response to water deprivation	40	5.9	412	1.66	5.62E-09
GO:0009415	response to water stimulus	40	5.9	420	1.69	9.72E-09
GO:0019748	secondary metabolic process	54	7.9	711	2.87	1.70E-08
GO:0032870	cellular response to hormone stimulus	56	8.2	762	3.07	2.42E-08
GO:0071495	cellular response to endogenous stimulus	56	8.2	763	3.08	2.43E-08
GO:0009755	hormone-mediated signaling pathway	54	7.9	721	2.91	2.50E-08
GO:0009738	abscisic acid mediated signaling pathway	27	4.0	213	0.86	3.18E-08
GO:0071215	cellular response to abscisic acid stimulus	28	4.1	230	0.93	3.51E-08
GO:0006633|GO:0000037	fatty acid biosynthetic process	29	4.3	250	1.01	4.98E-08
GO:0042538	hyperosmotic salinity response	22	3.2	162	0.65	4.76E-07
GO:0003824	catalytic activity	254	37.2	6722	27.11	1.92E-06
GO:0009737	response to abscisic acid stimulus	44	6.5	594	2.40	1.96E-06
GO:0016053	organic acid biosynthetic process	64	9.4	1063	4.29	2.26E-06

[a]Each GO category was significantly enrichment in the NMN-regulated gene by a Fisher's exact test (Corrected p-value).

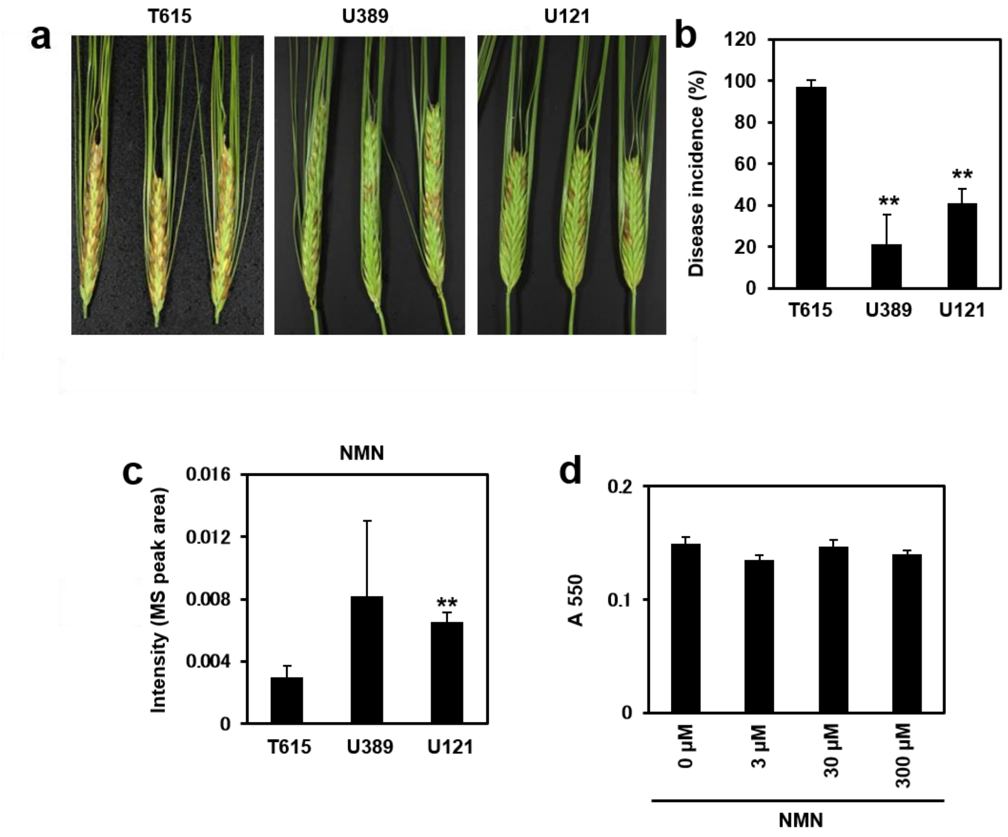

Supplementary Figure 1. NMN significantly accumulated in the FHB-resistant barley cultivars. (a) Representative photographs of *F. graminearum*-inoculated spikes of FHB-susceptible (T615; Turkey 45) and -resistant cultivar (U389; Maja, U121; Sirius O-525). (b) Disease incidence (color change of inoculated grains) was measured in T615, U389, and U121. Error bars represent the standard deviation (n=3). (c) NMN accumulation of T615, U389 and U121 cultivars without inoculation of *F. graminearum.* (d) Conidia of *F. graminearum* (1×10^3 conidia/ml) were cultured for 2 days in SN liquid medium. NMN (0, 3, 30, and 300 μM) was added to the medium. The growth of *Fusarium* was measured by 3-(4,5-di-methylthiazol-2-yl)-2,5-diphenyltetrazollium bromide (MTT) assay. Error bars represent the standard deviation (n=3). (Student's t-test **: P<0.01).

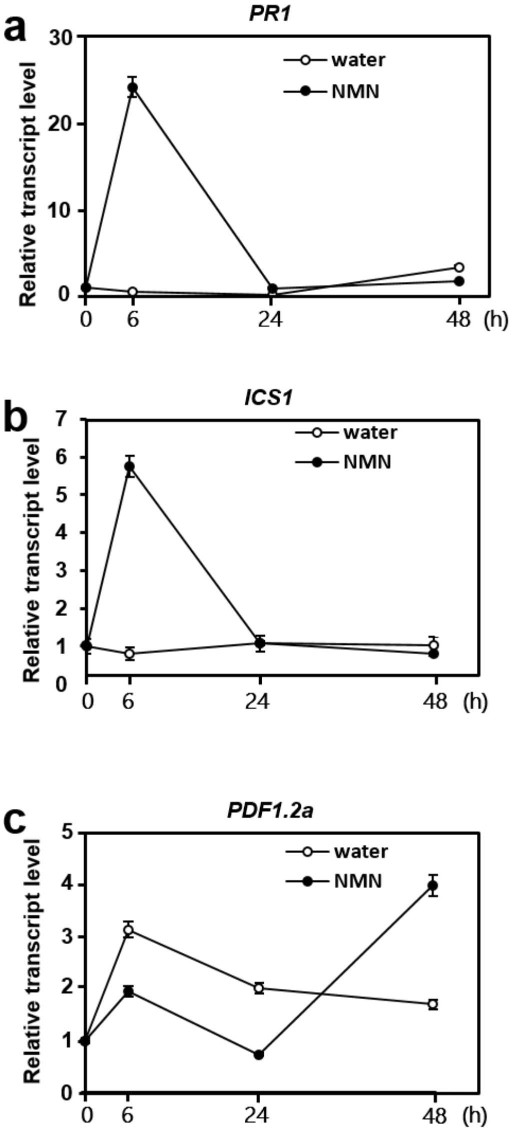

Supplementary Figure 2. NMN induces expression of the *PR1* and *ICS1* gene in Arabidopsis leaves. RT-qPCR analysis of *PR1*, *ICS1* and *PDF1.2a* gene in Arabidopsis leaves (Col-0) with and without NMN treatment. Tissues were harvested at 0, 6, 24, 48 h after spraying of NMN or water (0.001% silwetL77). *ACTIN2/8* (*Act2/8*) was used as the reference gene. Each value was shown as fold change (each sample vs 0h of water treatment). Data represent the mean of triplicate experiments ±S.D. (a, b) *PR1* and *ICS1* mRNA was transiently increased by NMN treatment. (c) NMN treatment did not induce expression of the *PDF1.2a* gene (n=3).

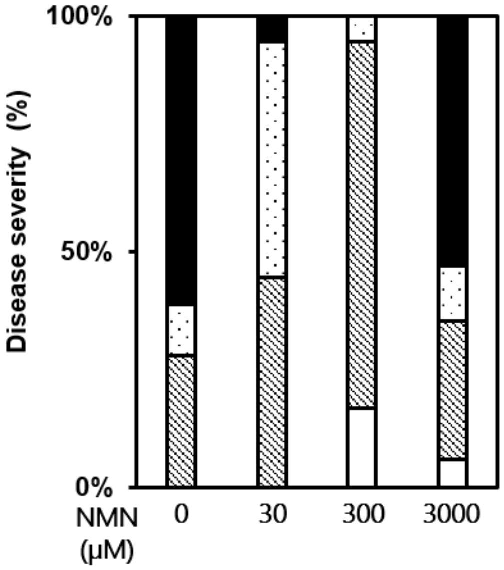

Supplementary Figure 3. Optimal concentration of NMN-induced disease resistance against *F. graminearum* in Arabidopsis. NMN at different concentrations was sprayed onto the surface of Arabidopsis rosette leaves prior to fungal incubation. After 6h of spraying, conidia solutions (1×10^5 conidia/ml) of *F. graminearum* were then injected into leaves. Disease severity of *F. graminearum*-inoculated leaves in each concentration. Disease severity was evaluated from observations of symptoms on inoculated leaves 3 days after inoculation (n=17-18). Open box: normal, cross-hatched box: colour change, dot box: partial aerial mycelium, closed box: expanded aerial mycelium.

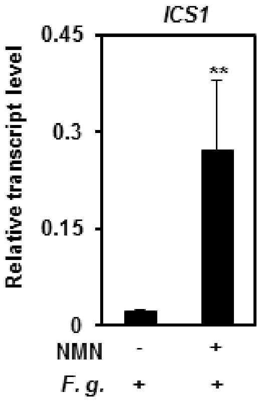

Supplementary Figure 4. Expression patterns of *ICS1* gene in *F. graminearum*-infiltrated Arabidopsis leaves with or without NMN pretreatment. RT-qPCR analysis of *ICS1* in Arabidopsis leaves with and without NMN pretreatment. After 6 h NMN pretreatment, leaves were injected with *F. graminearum* (*F.g.*). Inoculated plants were kept under high humidity conditions for 3 days. *ACTIN2/8* (*Act2/8*) was used as the reference gene. Error bars represent the standard deviation (n=3) (Student's t-test **: P<0.01).

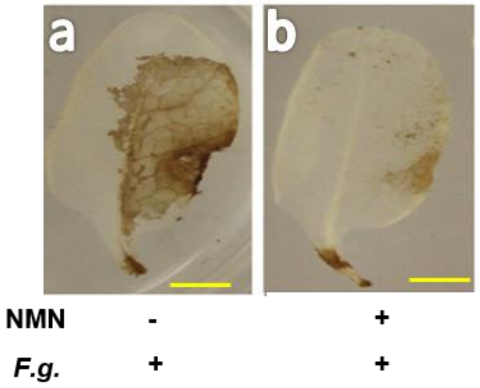

Supplementary Figure 5. NMN induced disease resistance against *F. graminearum* in Arabidopsis. NMN (300 μM) or water was sprayed onto the surface of Arabidopsis rosette leaves. After 6 h of spraying, conidia solutions (1×10^5 conidia/ml) of *F. graminearum* were then injected into leaves. Plants inoculated with *F. graminearum* were kept under high humidity conditions at 3 dpi. Representative photographs of 3,3-diaminobenzidine (DAB) stains of *F. graminearum*-inoculated leaves without (a) or with NMN pretreatment (b). Scale bars: 1 cm.

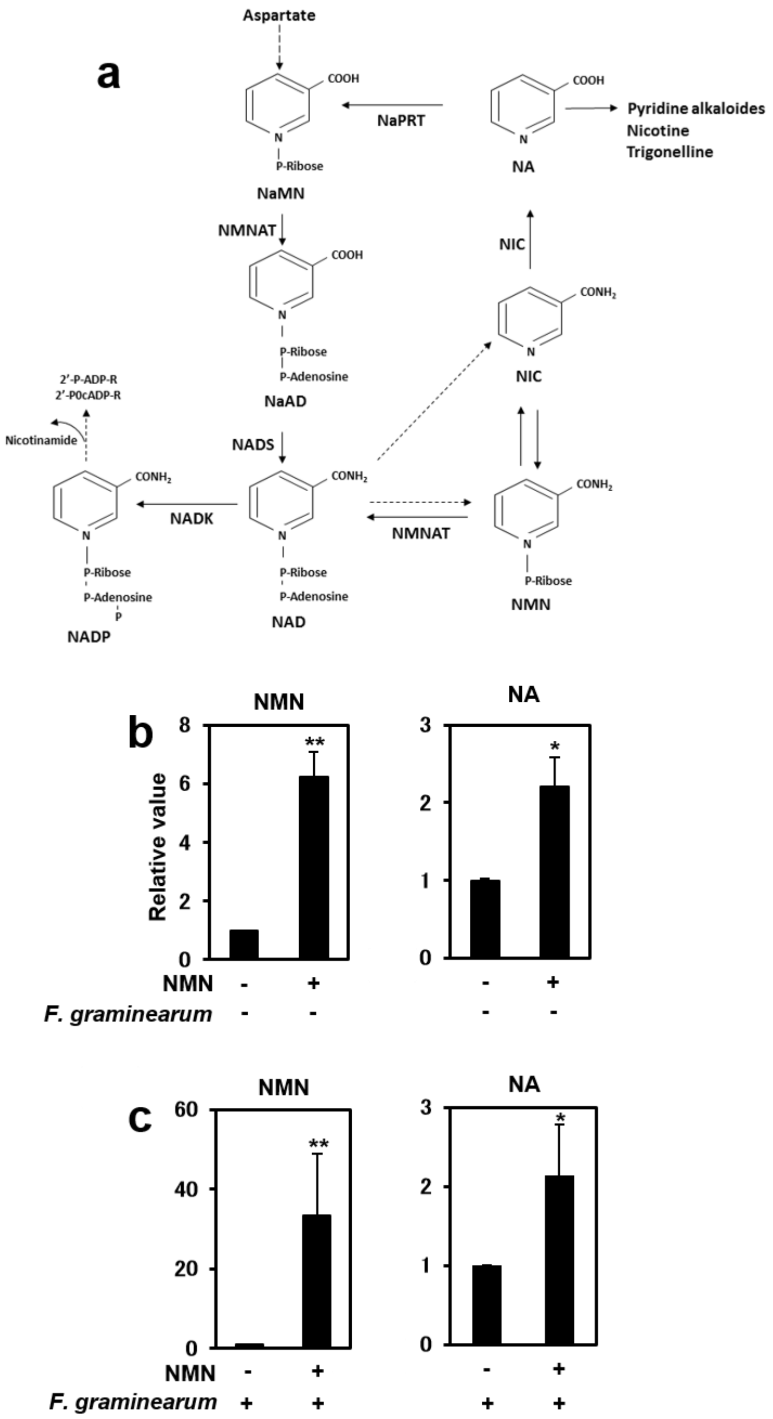

Supplementary Figure 6. Metabolome profiling of NMN-treated Arabidopsis leaves. (a) The diagram of the NAD biosynthesis pathway was modified[41]. Arrows indicate NAD biosynthesis pathway and dashed arrows indicate NAD degradation.

Supplementary Figure 6, (continued)

Abbreviations: NaMN; nicotinate mononucleotide, NaAD; nicotinate adenine dinucleotide, NAD; nicotinamide adenine dinucleotide, NMN; nicotinamide mononucleotide, NADP; nicotinamide adenine dinucleotide phosphate, NIC; nicotinamide, NA; nicotinate *NMNAT*; nicotinate/nicotinamide mononucleotide adenyltransferase, *NADS*; NAD synthetase, *NIC*; nicotinamidase, *NaPRT*; nicotinate phosphoribosyltransferase, *NADK*; NAD kinase. (b) NMN and NA content in uninoculated leaves. NMN or water were sprayed onto the surface of Arabidopsis rosette leaves. Tissues were harvested at 6 h after spraying. (c) NMN and NA content in inoculated leaves. NMN was sprayed onto the surface of Arabidopsis rosette leaves. After 6 h NMN treatment, conidia solutions (1×10^5 conidia/ml) of *F. graminearum* were injected into leaves. Tissues were harvested leaves 3 days after inoculation. Accumulation of NMN, NA, was quantified (n=3). (Student's t-test *: $P<0.05$ **: $P<0.01$).

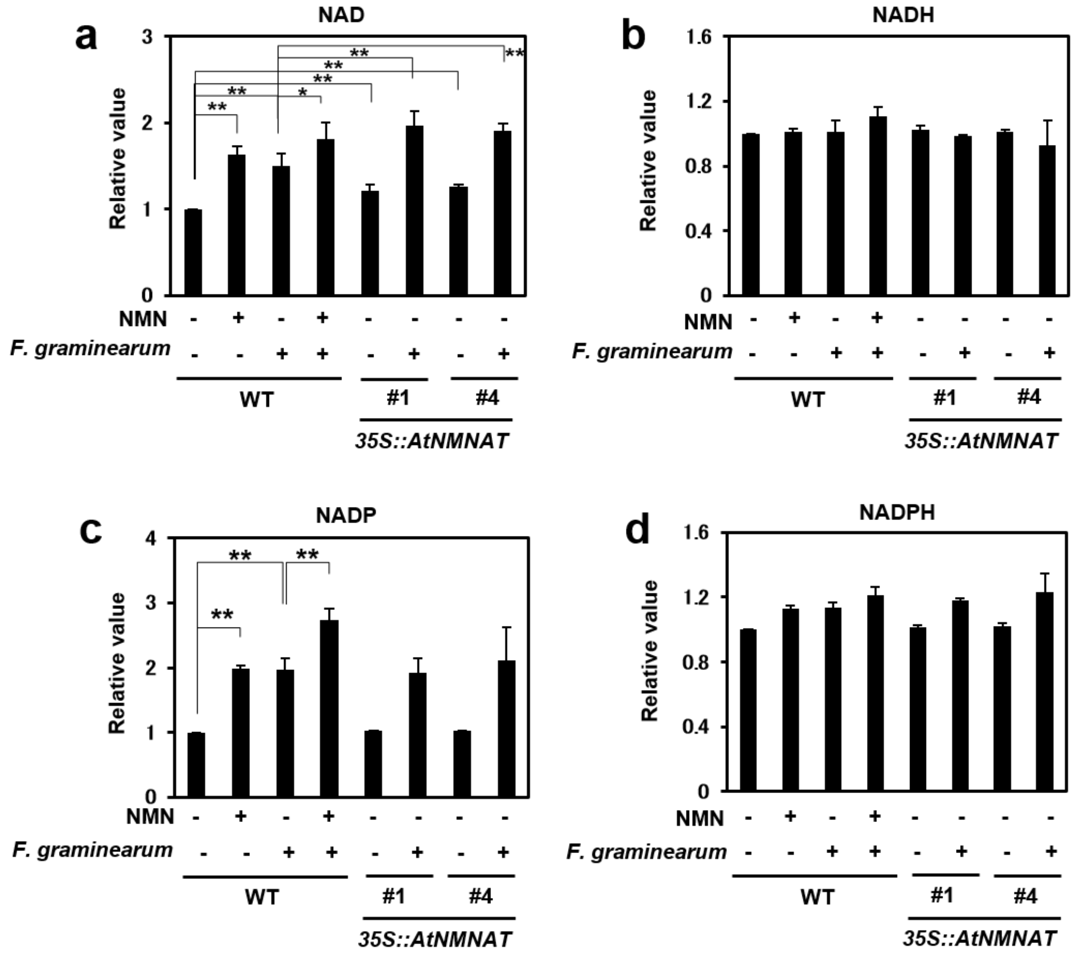

Supplementary Figure 7. NMN treatment and inoculation of *F. graminearum* induce increased NAD and NADP levels in Arabidopsis leaves. NAD(H) and NADP(H) levels in mature leaves of wt with and without NMN treatment were quantified. After 6 h incubation, the same leaves of wt were injected with water or conidia of *F. graminearum*. NAD(H) and NADP(H) levels were also quantified in leaves of two independent *35S::AtNMNAT* lines with and without inoculation of of *F. graminearum*. Error bars represent the standard deviation (n=5). (Student's t-test *: P<0.05 **: P<0.01).

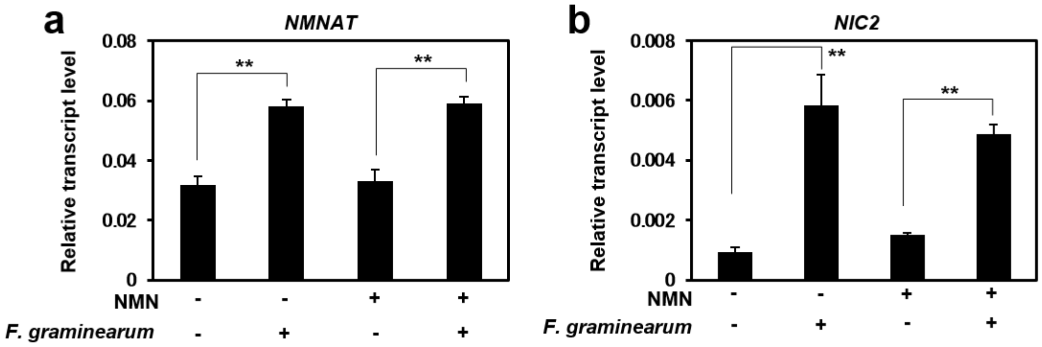

Supplementary Figure 8. Expression pattern of NAD biosynthetic genes by NMN application and inoculation of *F. graminearum*. RT-qPCR analysis of *NMNAT* and *NIC2* in Arabidopsis plants treated with and without NMN. After 6 h incubation, the same leaves were injected with *F. graminearum*. Error bars represent the standard deviation (n=3). Abbreviations: *NMNAT*, Nicotinate/nicotinamide mononucleotide adenyltranseferase; *NIC2*, Nicotinamidase 2. (Student's t-test, **: P<0.01).

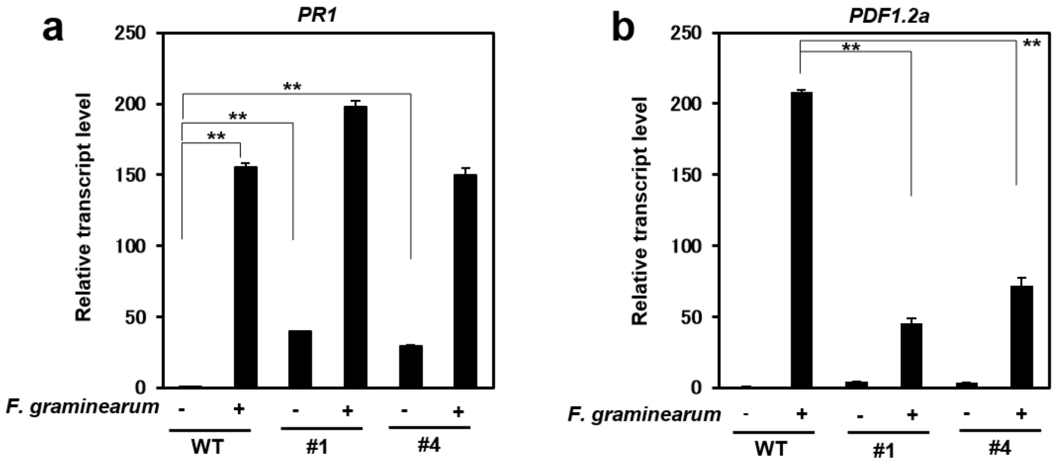

Supplementary Figure 9. Expression patterns of *PR1* and *PDF1.2a* genes in *F. graminearum*-infiltrated transgenic plants (*35S::AtNMNAT*) leaves. (a, b) RT-qPCR analysis of *PR1* and *PDF1.2a* in transgenic plants leaves were injected with and without *F. graminearum*. Inoculated plants were kept under high humidity conditions for 3 days. *ACTIN2/8* (*Act2/8*) was used as the reference gene. Each value was shown as fold change (each sample vs mock-treated WT). Error bars represent the standard deviation (n=3). (Student's t-test **: P<0.01).
